# Circulating Short-Chain Fatty Acid Profile Predicts Functional Outcome After Moderate-to-Severe Traumatic Brain Injury

**DOI:** 10.1097/CCE.0000000000001433

**Published:** 2026-06-11

**Authors:** Ori J. Lieberman, Sanhita Nittala, Luisa Rojas-Valencia, Sergio E. Baranzini, Neel S. Singhal, J. Claude Hemphill, Edilberto Amorim, Adam R. Ferguson, H. E. Hinson

**Affiliations:** 1 Department of Neurology, University of California San Francisco, San Francisco, CA.; 2 Weill Institute for Neuroscience, University of California San Francisco, San Francisco, CA.; 3 Brain and Spinal Injury Center, Zuckerberg San Francisco General Hospital, San Francisco, CA.; 4 Department of Neurological Surgery, University of California San Francisco, San Francisco, CA.; 5 San Francisco Veterans Affairs Healthcare System, San Francisco, CA.

**Keywords:** microbiome, neurocritical care, prognostication, short-chain fatty acids, traumatic brain injury

## Abstract

**OBJECTIVES:**

Short-chain fatty acids (SCFAs) are immunometabolites produced by the gut microbiome. In animal models, SCFAs affect traumatic brain injury (TBI) severity by modulating the immune response and serving as an energy source. The goal of this study was to assess whether SCFAs are associated with functional outcome in adult patients with moderate-to-severe TBI (msTBI).

**DESIGN:**

Prospective cohort study.

**SETTING:**

Urban Trauma Center.

**PATIENTS:**

Adults (age ≥ 15 yr) who had TBI with Glasgow Coma Scale 3–12, intracranial hemorrhage on head CT scan, and at least one reactive pupil. Blood samples had to be collected within 3 hours of trauma.

**INTERVENTIONS:**

None.

**MEASUREMENTS AND MAIN RESULTS:**

Univariate and multivariate analyses demonstrated that plasma SCFAs were associated with better functional outcomes at discharge and 6 months, an association driven primarily by differences in plasma acetate and propionate. K-means clustering of acetate and propionate levels identified two patient clusters with distinct discharge and 6-month functional outcomes but similar clinical, biomarker, and radiographic injury severity. Cluster 1 (*n* = 47) had higher SCFA levels compared with cluster 2 (*n* = 76) and cluster 1 had more favorable outcomes at discharge (Glasgow Outcome Scale 4–5: 83% vs. 55%; *p* = 0.003) and 6 months (Extended Glasgow Outcome Scale 4–8: 78% vs. 45%; *p* = 0.005). Multivariable logistic regression adjusting for the International Mission for Prognosis and Analysis of Clinical Trials in TBI (IMPACT)_lab_ model identified an independent association between the SCFA cluster and functional outcome at discharge (*p* = 0.001) and 6 months (*p* = 0.03). Adding the SCFA cluster to the IMPACT_lab_ model improved the area under the receiver operating characteristic curve for the prediction model for a favorable outcome.

**CONCLUSIONS:**

Our study suggests that SCFA levels are associated with functional outcome after msTBI. Future studies will focus on identifying mechanisms through which SCFAs may improve msTBI outcomes and what drives interpatient variation in their levels, which could position SCFAs as prognostic biomarkers and therapeutic targets in TBI.

**KEY POINTS**

**Question**: Do circulating levels of short-chain fatty acids (SCFAs) measured shortly after moderate-to-severe traumatic brain injury (msTBI) predict functional outcome?

**Findings**: Higher levels of SCFAs measured within 3 hours of msTBI are associated with more favorable outcomes, independently of primary injury severity. Adding SCFA measurements to existing prognostic scores improved their performance.

**Meaning**: Measuring blood SCFA levels may improve outcome prognostication after msTBI. Boosting SCFA levels after msTBI may be a novel therapeutic target to improve outcomes in this critically ill population.

Moderate-to-severe traumatic brain injury (msTBI) is common and highly morbid (1). Inflammation and neuronal energy failure are thought to contribute to secondary brain injury after msTBI and to worsen outcomes (2). Identifying potential modulators of secondary brain injury is a priority in neurotrauma research.

Short-chain fatty acids (SCFAs) are potent immunometabolic modulators primarily produced from insoluble dietary fiber by gut bacteria (3). Acetate, propionate, and butyrate are the primary SCFAs present in the peripheral blood. After their synthesis, SCFAs can be used either as an energy source (4) or as a signal through cognate G protein-coupled receptors, which are expressed by innate and adaptive immune cells as well as the endothelium (3). Circulating SCFAs bias brain, gut, and circulating innate and adaptive immune cells toward an anti-inflammatory phenotype (5–10). Animal models suggest that SCFA production is diminished after traumatic brain injury (TBI), and outcomes might be improved by SCFA supplementation (5–7, 9, 11). Furthermore, gut microbiome composition changes early after TBI in rodent models (6, 12–16) as well as in humans (17–19), and contributes to worsened neurologic outcomes via altered systemic and neuroinflammation and hippocampal neurogenesis. Whether SCFAs are similarly associated with outcome after TBI in humans remains unknown.

We hypothesized that higher circulating levels of SCFAs would be associated with better functional outcomes after msTBI. To assess this, we measured SCFAs in hyperacute plasma samples obtained from an observational, prospective cohort of msTBI patients. We subsequently assessed whether this association was dependent on initial injury severity and improved outcome prognostication compared with existing clinical scores, such as the International Mission for Prognosis and Analysis of Clinical Trials in TBI (IMPACT)_lab_ model (20) (**Supplemental Introduction**, https://links.lww.com/CCX/B642).

## METHODS

### Participants and Biomarkers

Participants were enrolled in the Predictors of Low-risk Phenotypes after Traumatic Brain Injury Incorporating Proteomic Biomarker Signatures (PROTIPS) study, details of which have been previously described (21). In brief, patients presenting with Glasgow Coma Scale (GCS) 3–12 and hemorrhage on the initial head CT scan after injury were eligible. Plasma was collected from subjects within 3 hours of injury, aliquoted, and frozen at –80°C for batch processing and analysis. Approval for the study was obtained from the Institutional Review Board at Oregon Health and Sciences University on May 23, 2023 (STUDY00021297), and the study required patient consent. All procedures were followed in accordance with the ethical standards of the responsible committee on human experimentation and with the Helsinki Declaration of 1975.

Samples from 123 of the 126 subjects in PROTIPS cohort were analyzed in the current study (three had insufficient volume for this assay and were excluded). The GOS at hospital discharge and the Extended GOS (GOSE) at 6 months were measured by trained research assistants. Discharge GOS 4–5 and 6-month GOSE 4–8 were considered favorable outcomes. Due to loss to follow-up, 35 enrolled subjects did not have available 6-month outcomes. In analyses where the outcome was 6-month GOSE, complete-case analysis was performed, and subjects lost to follow-up were excluded. Radiographic features were adjudicated by fellowship-trained neuroradiologists.

Protein blood-based biomarkers (glial fibrillary acidic protein [GFAP] and neurofilament light chain [NfL]) were measured using an electrochemiluminescence immunoassay in an array-based multiplex format (MesoScale Diagnostics, Rockville, MA). SCFA levels were quantified using gas chromatography mass spectrometry (GC-MS), as previously described (22). As in our previous study (22), samples underwent a single freeze-thaw cycle before GC-MS for detection of SCFAs. NfL, GFAP, and SCFA levels were measured in the same sample collected within 3 hours of initial injury.

### Statistical Analysis

Type 1 error rate was set at 0.05 for statistical significance. The association between SCFA levels and favorable outcomes were determined using both univariate and multivariate analyses. Two-way analysis of variance (ANOVA) and multivariable logistic regression were used to determine the univariate association between SCFAs and outcome. Appropriate post hoc tests were used to determine the statistical significance of specific SCFAs between outcome groups, as detailed in the *Results* and *Figure Legends*. To overcome limitations surrounding the collinearity of SCFAs in human plasma (22), which may confound univariate analyses (23, 24), multivariate analysis using partial least square discriminant analyses (PLS-DA)—details of which are included in the **Supplemental Methods** (https://links.lww.com/CCX/B642) (25)—was performed in parallel. Variable Importance in Projection (VIP) scores were calculated for each SCFA, and those with VIP scores greater than 1 were considered associated with favorable outcome and selected as features for downstream analyses (24).

To define the relationship between injury severity and SCFA levels, a staged clustering approach was employed. K-means clustering was performed using SCFAs that were significantly associated with outcome in the PLS-DA models. The optimal cluster number was selected using the silhouette score and cluster robustness was assessed using the between-cluster sum-of-squares to total sum-of-squares ratio (BSS/TSS). In line with the recently proposed clinical-biomarker-imaging modifier (CBI-M) framework, baseline characteristics as well as clinical, blood-based biomarkers (NfL and GFAP), and imaging characteristics of TBI severity were assessed between SCFA clusters, using the Mann-Whitney *U* test or the Kruskal-Wallis test as applicable (26). The functional outcome at discharge and 6 months between clusters was compared using the chi-square test or ordinal regression.

Logistic regression models were developed to assess whether plasma SCFA levels were independently associated with outcome by adjusting for the predicted probability of an unfavorable outcome derived from the IMPACT_lab_ model, which is a well-validated clinical prediction tool, including in the PROTIPS cohort (21), for a favorable outcome in TBI patients (20, 27–29). Parallel analyses were performed limiting the analysis to SCFAs significantly associated with outcome in the univariate and multivariate analyses.

Model performance, including SCFA levels or SCFA cluster, was evaluated using the likelihood-ratio test and the area under the receiver operating characteristic curve (AUROC). To determine how the predicted risk of unfavorable outcome changed with the addition of the SCFA cluster to the prediction model, the baseline risk of unfavorable outcome from the IMPACT_lab_ model was subtracted from the risk of unfavorable outcome determined by a combined model that included both IMPACT_lab_ and SCFA cluster. To optimize power for these analyses, only the discharge outcome was used. The differences in predicted risk based on SCFA cluster as a function of the probability of the IMPACT_lab_ model were compared using linear regression and across IMPACT_lab_ strata using two-way ANOVA with Sidak’s multiple comparisons test. Finally, five-fold stratified cross-validation was used to generate the predicted probabilities of unfavorable outcomes in the combined SCFA cluster and IMPACT_lab_ model, and the above analyses were repeated with the out-of-fold predicted probability of unfavorable outcome (Supplemental Methods, https://links.lww.com/CCX/B642).

## RESULTS

### Plasma SCFA Levels Are Associated With Discharge and 6-Month Functional Outcome After msTBI

**Figure 1.**
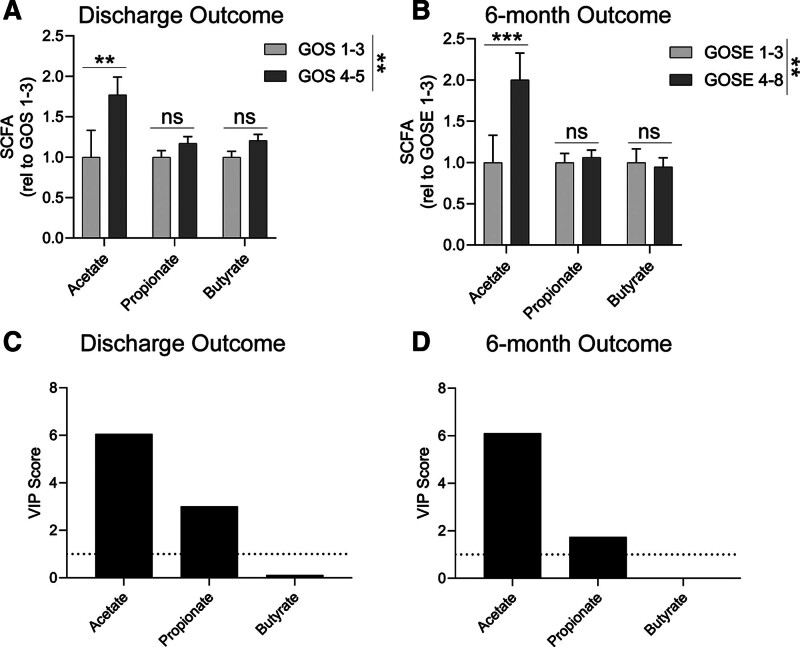
Hyperacute plasma short-chain fatty acid (SCFA) levels are associated with discharge and 6-mo functional outcome after moderate-to-severe traumatic brain injury. **A**, SCFA levels normalized to the Glasgow Outcome Scale (GOS) 1–3 group demonstrate a significant main effect of outcome group and post hoc Holm-Sidak testing revealed that acetate was significantly different between outcome groups. Two-way analysis of variance (ANOVA): SCFA × outcome group (*F*_(2, 363)_ = 1.877; *p* = 0.15); SCFA (*F*_(2, 363)_ = 1.877; *p* = 0.15); and outcome group: (*F*_(1, 363)_ = 7.324; *p* = 0.0071). **B**, SCFA levels normalized to the extended GOS (GOSE) 1–3 group demonstrate a significant interaction between outcome group and SCFA. Post hoc Holm-Sidak testing revealed that acetate was significantly different between outcome groups. Two-way ANOVA: SCFA × outcome group (*F*_(2, 172)_ = 3.631; *p* = 0.0279). **C** and **D**, Variable Importance in Projection (VIP) score for log-transformed, scaled SCFAs determined using partial least square discriminant analyses (PLS-DA) with favorable outcome at discharge as the dependent variable (*n* = 123; **C**) and at 6 mo was used as the dependent variable (*n* = 88; **D**) in PLS-DA. **p < 0.01, ***p < 0.001. ns = not significant.

The demographics of the included subjects from the PROTIPS study are shown in **Table 1**, and the TBI characteristics are shown in **Table 2**. Seven SCFA species were detected at varying levels in plasma, with acetate, propionate, and butyrate being the most abundant and highly correlated within subjects (**Fig. 1**, ***A*** and ***B*** and **eFig. 1**, https://links.lww.com/CCX/B642). SCFA levels did not differ between subjects with ascertained or missing 6-month outcomes (**eFig. 2**, https://links.lww.com/CCX/B642), and there was no association between SCFA levels and the time elapsed between injury and blood collection (eFig. 1*I*, https://links.lww.com/CCX/B642). In univariate analyses, the SCFA, acetate, was significantly associated with functional outcomes at discharge and at 6 months (Fig. 1, *A* and *B*).

**TABLE 1.** Demographics of Predictors of Low-Risk Phenotypes After Traumatic Brain Injury Incorporating Proteomic Biomarker Signatures Cohort and Short-Chain Fatty Acid Clusters

Characteristic	Full Cohort (*n* = 123)	Cluster 1 (*n* = 47)	Cluster 2 (*n* = 76)	*p*
Age, yr, mean ± sd	46 ± 21	40 ± 17	49 ± 23	0.008^a^
Sex, male, %	71	72	70	0.84^b^
Race, %				0.26^b^
Asian	4	0	7	
Black	9	11	8	
Native American	4	4	0	
Native Hawaiian or Pacific Islander	2	2	1	
Other or decline to answer	7	9	5	
White	77	75	79	
Ethnicity, %				0.27^b^
Hispanic or Latino	7	11	5	
Not Hispanic or Latino	90	85	93	
Decline to answer	2	4	1	
Body mass index, mean ± sd	26.2 ± 6.7	26.6 ± 6.3	25.9 ± 6.9	0.56^a^

a Two-tailed unpaired *t* test.

b χ^2^ test.

**TABLE 2.** Characteristics of Injury Severity in the Predictors of Low-Risk Phenotypes After Traumatic Brain Injury Incorporating Proteomic Biomarker Signatures Cohort and Short-Chain Fatty Acid Clusters

Characteristics	Full Cohort (*n* = 123)	Cluster 1 (*n* = 47)	Cluster 2 (*n* = 76)	*p*
Glasgow Coma Scale at enrollment, median (IQR)	7 (6–10)	8 (6–10)	7 (4–10)	0.73^a^
Head AIS, median (IQR)	4 (3–5)	4 (3–5)	4 (3–5)	0.77^a^
Total body AIS, median (IQR)	23 (16.75–29)	21 (14–29)	24 (17–29)	0.32^a^
Pupillary reactivity, %				0.20^b^
Neither	6	9	4	
One	15	9	18	
Both	80	83	78	
Hypoxia present, %	8	13	5	0.25^b^
Hypotension present, %	14	13	15	1.00^b^
Rotterdam score, median (IQR)	2 (2–3)	2 (2–3)	2 (2–3)	0.98^a^
Marshall score, median (IQR)	2 (2–3)	2 (2–3)	2 (2–3)	0.76^a^
Traumatic subarachnoid hemorrhage present, %	95	96	95	1.00^b^
Epidural present, %	3	6	1	0.31^b^
Glucose (mg/dL), mean ± sd	150 ± 54.4	139 ± 50.1	156 ± 56.2	0.093^a^
Hemoglobin (g/dL), mean ± sd	13.1 ± 1.8	13.6 ± 1.5	12.8 ± 1.9	0.023^c^
Glial fibrillary acidic protein (log_10_ pg/mL), mean ± sd	6.43 ± 0.72	6.35 ± 0.64	6.48 ± 0.77	0.25^c^
Neurofilament light chain (log_10_ pg/mL), mean ± sd	5.54 ± 0.57	5.44 ± 0.43	5.60 ± 0.64	0.11^c^

AIS = Abbreviated Injury Scale, IQR = interquartile range.

a Mann-Whitney *U* test.

b χ^2^ test.

c Two-tailed unpaired *t* test.

We employed PLS-DA to investigate the relationship between functional outcomes after msTBI and levels of the SCFAs acetate, propionate, and butyrate (**Fig. 1**, ***C*** and ***D*** and Supplementary Methods, https://links.lww.com/CCX/B642). The AUROC for the PLS-DA model was 0.68 for a favorable outcome at discharge and 0.67 for a favorable outcome at 6 months. VIP scores were greater than 1 for acetate and propionate in both models, suggesting a significant association with favorable outcomes, and these SCFAs were selected for further analyses (Fig. 1, *C* and *D*).

### SCFA Cluster Is Associated With Discharge and 6-Month Functional Outcome After msTBI Independently of Injury Severity

**Figure 2.**
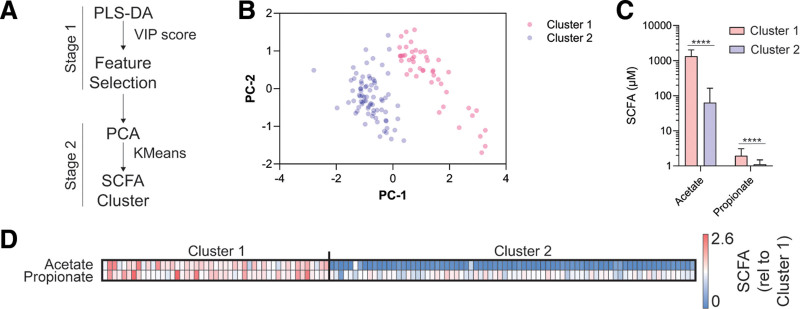
K-means clustering reveals two patient groups based on their short-chain fatty acid (SCFA) profile. **A**, Analysis pipeline that combines supervised and unsupervised approaches to identify patient clusters based on SCFA profile. **B**, Principal component analysis (PCA) of SCFAs with Variable Importance in Projection (VIP) score greater than 1 (acetate and propionate) was performed, followed by K-means clustering (k = 2) based on the PCA coordinates. *Each dot* represents a patient and *dots* are *colored* by K-means cluster number. **C** and **D**, Plasma acetate and propionate are significantly higher in cluster 1. **C**, Median and interquartile range. Two-way analysis of variance: SCFA × cluster (*F*_(1, 242)_ = 259.7; *p* < 0.0001). ****p < 0.0001 Holm-Sidak post hoc test. **D**, Heat map of acetate and propionate normalized to the mean for cluster 1. *Each column* represents a single subject. PC-1 = principal component-1, PC-2 = principal component-2, PLS-DA = partial least square discriminant analyses.

**Figure 3.**
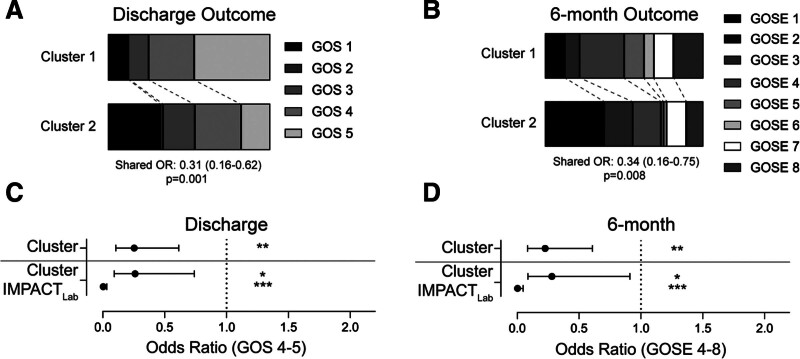
Short-chain fatty acid (SCFA) cluster is independently associated with functional outcome at discharge and at 6 mo after moderate-to-severe traumatic brain injury. **A** and **B**, Shift analysis of outcomes at discharge (**A**) and at 6 mo (**B**) between SCFA cluster. Ordinal regression revealed a significant effect of SCFA cluster across strata. Shared odds ratio (OR) with 95% CI and *p* value shown for each analysis. **C** and **D**, Logistic regression was performed with functional outcome at discharge (**C**) or 6 mo (**D**). Cluster assignment (reference = cluster 1) was significantly associated with functional outcome at discharge (**C**) and 6 mo (**D**; above *horizontal line*), even after adjustment for the International Mission for Prognosis and Analysis of Clinical Trials in TBI (IMPACT)_lab_ model (below *horizontal line*). *Dots* show OR and *error bars* show 95% CI. *p < 0.05, **p < 0.01, ***p < 0.001. GOS = Glasgow Outcome Scale, GOSE = Extended Glasgow Outcome Scale.

Acetate and propionate levels for each subject underwent dimensionality reduction with principal component analysis, and then K-means clustering was performed, with k = 2 yielding the highest silhouette score (**Fig. 2**, *A* and ***B*** and **eFig. 3**, https://links.lww.com/CCX/B642; silhouette score_k = 2_ = 0.54). These clusters demonstrated substantial separation in feature space (BSS/TSS = 0.55), and this separation was not fully explained by outcome alone (adjusted rand index [ARI] = 0.02 and normalized mutual information [NMI] = 0.07), suggesting these clusters capture distinct metabolic groups. Cluster 1 (*n* = 47) had higher acetate and propionate levels, compared with cluster 2 (*n* = 76; **Fig. 2**, ***C*** and ***D***). Subjects assigned to SCFA cluster 1 had more favorable outcomes at discharge (GOS 4–5: 83% vs. 55%; *p* = 0.003) and 6 months (GOSE 5–8: 78% vs. 45%; *p* = 0.005). Ordinal regression revealed that the effect of SCFA cluster assignment was present across GOS and GOSE strata (**Fig. 3**, ***A*** and ***B***). Patient characteristics and injury severity were compared between clusters using the CBI-M framework (26). Although age was significantly higher in cluster 2 (median [interquartile range]: cluster 1: 38 yr [25–52 yr] and cluster 2: 47 yr [27.5–67 yr]), there was no difference in the enrollment GCS, head Abbreviated Injury Scale (AIS) (30), total body AIS, or proportion of patients with unreactive pupils (Tables 1 and 2). Similarly, there were no differences in the Rotterdam or Marshall scores on the initial CT scan (Table 2). No difference was observed in the level of GFAP or NfL on emergency department arrival between SCFA clusters (Table 2).

Assignment to SCFA cluster 2 was significantly associated with lower odds of a favorable outcome at discharge (odds ratio [OR], 0.25; 95% CI, 0.11–0.61; *p* = 0.002) and at 6 months (OR, 0.23; 95% CI, 0.084–0.61; *p* = 0.003; **Fig. 3**, ***C*** and ***D***, above horizontal line). In a multivariable model that included the unfavorable outcome probability from the IMPACT_lab_ model (Supplemental Methods, https://links.lww.com/CCX/B642), which has been previously validated in the PROTIPS cohort (21) and incorporates age, assignment to SCFA cluster 2 was associated with a significantly lower odds of favorable outcome at discharge (adjusted OR [aOR], 0.26; 95% CI, 0.092–0.74; *p* = 0.001) and at 6 months (aOR, 0.28; 95% CI, 0.086–0.91; *p* = 0.03; Fig. 3, *C* and *D*, below horizontal line).

Sensitivity analyses were performed as follows. To ensure the independent association with SCFA levels with outcome was not an artifact of the K-means clustering algorithm, a logistic regression model was developed using principal component-1 (PC-1), which was associated with both acetate and propionate levels (**eFig. 4**, https://links.lww.com/CCX/B642), as a proxy for SCFA levels. PC-1 was significantly associated with a favorable discharge outcome (OR, 1.58; 95% CI, 1.098–2.27; *p* = 0.01) and remained so after adjustment for IMPACT_lab_ (aOR, 1.63; 95% CI, 1.06–2.50; *p* = 0.03). Because age was the only significantly different baseline characteristic between SCFA clusters, a logistic regression model was developed to predict a favorable outcome using SCFA cluster and adjusting for age as a continuous variable. Assignment to SCFA cluster 2 was significantly associated with lower odds of a favorable outcome at discharge after adjustment for age (aOR, 0.30; 95% CI, 0.12–0.77; *p* = 0.01). A separate model was constructed using acetate, the only SCFA that was significantly different across outcome groups in univariate analyses, by adjusting for either the predicted probability of unfavorable outcome in the IMPACT_lab_ model or age. Log_10_-transformed acetate was associated with a favorable outcome at discharge after msTBI after adjustment for the IMPACT_lab_ model (aOR, 1.75; 95% CI, 1.09–2.83; *p* = 0.02) or age (aOR, 1.64; 95% CI, 1.07–2.50; *p* = 0.02). Together these results suggest a robust, independent association between SCFAs and outcome after msTBI.

### SCFA Cluster Asymmetrically Improves Risk Prediction Across the Spectrum of Injury Severity in the PROTIPS Cohort

**Figure 4.**
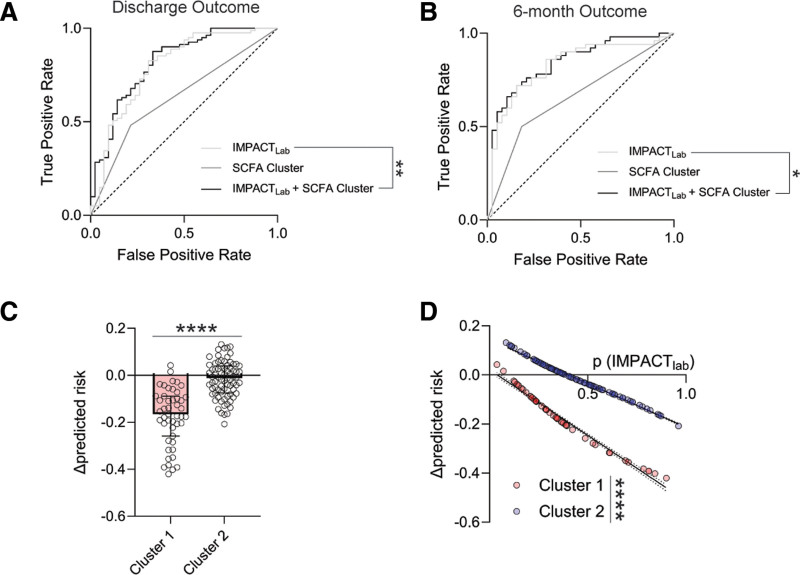
Addition of short-chain fatty acid (SCFA) cluster to the International Mission for Prognosis and Analysis of Clinical Trials in TBI (IMPACT)_lab_ outcome prediction model improves model performance and identifies a subgroup of patients with an overestimated predicted probability of unfavorable outcome. **A** and **B**, Receiver operating characteristic curves for logistic regression models including cluster assignment, IMPACT_lab_, or both as predictors for outcome at discharge (**A**) or at 6 mo (**B**). Discharge: (SCFA cluster + IMPACT_lab_ vs. IMPACT_lab_ likelihood-ratio test; *p* = 0.008) and 6 mo: (SCFA cluster + IMPACT_lab_ vs. IMPACT_lab_; *p* = 0.03). **C**, The change in predicted risk of unfavorable outcome between the outcome prediction model using both SCFA cluster + IMPACT_lab_ or using IMPACT_lab_ alone (Δpredicted risk) was greater in subjects assigned to SCFA cluster 1. Two-tailed unpaired *t* test (*p* < 0.0001). **D**, The relationship between Δpredicted risk and the baseline probability of an unfavorable outcome from the IMPACT_lab_ model is different between SCFA clusters. Analyzed using linear regression, which showed that slopes were significantly different between SCFA clusters (*p* < 0.0001). *p < 0.05, **p < 0.01, ****p < 0.0001.

We next sought to determine whether adding SCFA cluster assignment to existing prediction models (IMPACT_lab_) improved outcome prediction after msTBI. Addition of SCFA cluster assignment to the IMPACT_lab_ model improved the prediction of discharge (**Fig. 4*A***; AUROC_SCFA + IMPACT_ 0.83 vs. AUROC_IMPACT_ 0.80; *p* = 0.008) and 6-month outcome (**Fig. 4*B***; 0.84 vs. 0.83; *p* = 0.03). Interestingly, we found that the difference in predicted risk of unfavorable outcome at discharge between the baseline IMPACT_lab_ model and a model that included both IMPACT_lab_ and SCFA cluster assignment differed by SCFA cluster (**Fig. 4*C***). Subjects assigned to SCFA cluster 1 had a larger decrease in predicted risk of unfavorable risk compared with SCFA cluster 2 in the combined model (Fig. 4*C*; cluster 1: –0.18 ± 0.017; cluster 2: –0.017 ± 0.0088; *p* < 0.0001). The difference in predicted risk between clusters was maximal in subjects with the highest predicted risk of unfavorable outcome based on IMPACT_lab_ alone (**Fig. 4*D***; ordinal least square regression: IMPACT_lab_ × SCFA cluster interaction *p* < 0.0001). Similar results were obtained using tertiles of predicted probabilities of unfavorable outcomes from IMPACT_lab_ (data not shown; two-way ANOVA IMPACT_lab_ tertile × SCFA cluster interaction *p* < 0.0001).

Five-fold stratified cross-validation was subsequently performed to assess the internal validity of these results. Stratified folds preserved the proportion of subjects with unfavorable outcomes. The updated probability of a favorable outcome for each subject was based on the combined SCFA cluster and IMPACT_lab_ model trained on a four-fold and evaluated on the held-out fold. Predicted probabilities from the held-out folds were used to generate out-of-fold predictions for each subject. The baseline predicted probabilities using IMPACT_lab_ alone were not refitted using cross-validation given the broadly accepted generalizability of this model (20, 21, 27–29). Similar results were seen using out-of-fold predictions (**eFig. 5**, https://links.lww.com/CCX/B642).

Together, these results suggest that the addition of an SCFA cluster to existing clinical outcome prediction models after msTBI improves model performance (Fig. 4, *A* and *B*) and that, among patients with a higher baseline risk of unfavorable outcome based on IMPACT_lab_, the SCFA cluster identified a subgroup of patients whose risk of unfavorable outcome is overestimated (Fig. 4, *C* and *D*).

## DISCUSSION

SCFAs are potent immunomodulatory metabolites produced primarily by the gut microbiome and are protective in animal models of TBI (5–7, 9, 11). We extend this finding to msTBI patients and, for the first time, demonstrate that higher SCFA levels measured in the hyperacute period after msTBI are associated with a favorable functional outcome at discharge and at 6 months. Furthermore, we show that the incorporation of SCFA levels into existing clinical outcome prediction scores identifies a subgroup of patients with an overestimated probability of an unfavorable outcome. These data suggest that SCFA levels after msTBI may serve as a prognostic biomarker for functional recovery and provide a potential therapeutic target to mitigate secondary neurologic injury.

The evidence supporting a protective role for SCFAs after TBI comes from rodent studies that demonstrated a reduction in plasma and fecal acetate levels after controlled cortical impact (5, 6) and that supplementation of SCFAs in drinking water (acetate, propionate, and butyrate) or via a modified diet (acetate and butyrate only) improves neurologic outcomes and brain pathology after injury (5, 7, 9). As such, SCFAs have been proposed to represent a mechanistic explanation for the contribution of the gut-brain axis to outcome after TBI (31–34). Here, we demonstrate that msTBI patients with lower SCFA levels at presentation to a level I trauma center had a higher risk of having an unfavorable outcome at discharge and at 6 months. In our dataset, both univariate and multivariate analyses identified acetate as significantly associated with outcome. In the multivariate PLS-DA analysis, a significant association between propionate and outcome was also seen. It remains unclear how strong the association between propionate and outcome is after msTBI in humans. Further studies in larger cohorts with serial SCFA measurements will allow for this association to be tested. However, our data, in addition to the animal experiments from several groups described above, identify an association between higher SCFA levels and better outcomes after TBI.

SCFA supplementation reduces ischemia burden in animal models of ischemic stroke, and SCFA levels are associated with better functional outcomes and reduced radiographic evidence of hypoxic-ischemic brain injury in human survivors of cardiac arrest (22, 35–38). After stroke and cardiac arrest, propionate and butyrate have been identified as the primary beneficial SCFAs. Together, this suggests that higher SCFA levels may be associated with better outcomes across different types of brain injury, but the specific SCFAs that mediate this effect may be brain injury specific.

K-means clustering was performed using acetate and propionate levels, the two SCFAs identified in multivariate analyses as being significantly associated with functional outcome, to determine whether distinct metabolic groups were present in our dataset. This clustering method was unsupervised; clustering was performed in the absence of outcome labels. Using the silhouette score, k = 2 was selected for the number of clusters. Interestingly, visual inspection of acetate levels suggested a bimodal distribution (eFig. 1, https://links.lww.com/CCX/B642), which may explain the strength of the clustering model as assessed by a silhouette score of 0.54 and a BSS/TSS of 0.55 (39, 40). The SCFA clusters were not merely a representation of outcome, as demonstrated by the low ARI and NMI, emphasizing that the SCFA clusters capture variation in SCFA levels as opposed to leakage of outcome into the clustering algorithm.

In the present study, we measured SCFA levels in hyperacute plasma samples collected within 3 hours of injury. At this timepoint, SCFA levels may reflect baseline differences in SCFA production or reflect the impact of injury severity on SCFA production. Our analyses suggest that injury severity did not impact SCFA levels measured at this timepoint (Fig. 1 and Table 2). By clustering patients based on their SCFA profile, we were able to demonstrate that patients with higher circulating levels of acetate and propionate were more likely to have a favorable outcome despite having similar injury severity characterized using the CBI-M framework (26). We confirmed this independent association between SCFA cluster and outcome with logistic regression by adjusting for the IMPACT_lab_ prediction score (20). Notably, age was the only demographic variable that was significantly different between SCFA clusters and was included as a variable in IMPACT_lab_. Adjustment for age alone did not affect the association between SCFA cluster and outcome group.

Instead, we hypothesize that the interpatient variability in hyperacute plasma SCFA levels may reflect either preinjury differences in nutrition (substrate availability), gut microbiome composition (synthesis capacity), intestinal and hepatic function (utilization before reaching the systemic circulation), or post-injury iatrogenic interventions that impact SCFA synthesis (e.g., antibiotic administration) (41–46). Additional studies will be needed to assess the relative contributions of each of these factors by prospectively collecting information from patients and relatives around the time of injury.

Whether SCFAs are therapeutic targets that could improve outcomes after msTBI or merely predictive biomarkers remains unknown. As mentioned above, animal models provide strong evidence that manipulation of SCFA levels after experimental TBI leads to improved neurologic outcome and the rescue of cellular and neuropathologic changes seen after injury (5, 7, 9). Higher circulating levels of acetate and propionate may affect the outcome after msTBI via immunomodulation (3), acting as an alternative energy source (4), or merely reflect differences in gut-microbiome composition that independently modulate TBI. Future studies will delineate these possibilities. Our study raises key questions about the design of future studies that could test the therapeutic efficacy of SCFA supplementation after TBI. First, consideration must be given to baseline interpatient differences in SCFA levels. Such a clinical trial could be enriched for patients with low baseline levels of SCFAs to optimize the chances of identifying a potential benefit from this intervention. Alternatively, SCFA levels may decline in the days after injury in all patients, and SCFA supplementation may benefit a broader population of TBI patients. Future studies should measure plasma SCFA levels serially to test this hypothesis. Second, the optimal timing of intervention remains unknown. Animal studies have supplemented SCFAs for weeks to months after TBI. Here, we show that ultraearly measurement of SCFA levels predicts outcome after msTBI, suggesting that hyperacute treatment with SCFAs may benefit patients. Serial measurements of SCFAs in msTBI patients will be important to delineate the trajectory of SCFAs after injury and to determine the optimal timing of any intervention. Third, our data suggest that having higher SCFA levels (being assigned to SCFA cluster 1) reduced the predicted risk of unfavorable outcomes differentially across baseline risk of unfavorable outcomes, with patients with higher predicted risk having the largest reduction in predicted risk if they are in the high SCFA cluster (Fig. 4). This perhaps suggests that future studies that test whether SCFA supplementation improves outcomes may need to focus on more severely injured TBI patients. As such, the data reported in the present study will inform further observational and interventional studies aimed at determining the optimal patient population and intervention to assess whether SCFA supplementation improves outcome after TBI.

At present, blood-based prognostic biomarkers of outcome after TBI have focused on measuring neuronal and glial injury, such as GFAP, NfL, Tau, and S100 calcium-binding protein B (47). Here, we have shown that measurement of metabolites produced by the gut microbiome are independently associated with functional outcome after msTBI. In fact, acetate levels were not correlated with blood GFAP and NfL levels measured from the same samples (data not shown and Table 2), highlighting the orthogonal prognostic information provided by plasma SCFA levels. Peripheral measurement of neuronal and glial-based biomarkers poses distinct challenges, as their blood levels depend not only on the severity of neuronal and glial injury but also on blood-brain barrier permeability and systemic clearance. The discrepancy between cerebrospinal fluid and blood measurement of these biomarkers has been highlighted in both the TBI and cardiac arrest literature (48–53). Our findings raise the possibility that blood-based biomarkers that correspond to peripheral modifiers of brain injury may add to multimodal prognostication algorithms and improve patient care.

Limitations of our study include being from a single center with a limited sample size, sampling at a single time point, exclusion of msTBI patients without intracranial hemorrhage, and loss to follow-up at 6 months. Limited information is available regarding preinjury neurologic comorbidities, which could independently impact functional recovery trajectory. Because SCFA synthesis is dependent on environmental factors, such as diet and gut microbiome composition, future studies in broadly representative populations should incorporate assessments of these variables to fully evaluate the generalizability of SCFAs as candidate biomarkers. Serial measurements of SCFAs after TBI will be crucial to understanding whether interventions in the ICU may further affect SCFA levels and confound their potential as prognostic biomarkers. Finally, whether SCFA levels may predict outcome after mild or uncomplicated TBI remains unknown.

## CONCLUSIONS

Here, we demonstrate that higher circulating levels of SCFAs measured immediately after msTBI are associated with better functional outcomes after injury. Future studies will investigate potential underlying mechanisms and further evaluate the potential of SCFAs as biomarkers and therapeutic targets to enhance outcomes after acute brain injury.

## Supplementary Material
