# Supplementary material for: Circulating Short-Chain Fatty Acid Profile Predicts Functional Outcome After Moderate-to-Severe Traumatic Brain Injury
Source: Crit Care Explor. 2026 Jun 11;8(6):e1433. doi: 10.1097/CCE.0000000000001433 (PMC13259564; doi:10.1097/CCE.0000000000001433)
Supplement: Supplementary file 1 [file cc9-8-e1433-s001.pdf]

Supplemental Introduction

The IMPACT model is a prognostic score for the prediction of mortality and unfavorable outcome after TBI. Its development has allowed for outcome prediction based on admission patient, injury, imaging and laboratory data, which has informed patient selection for clinical trials and prognostic discussions with patient families. It was developed from aggregated individual patient data accumulated across 11 clinical trials in the mid-2000's [1,2] and has been validated in modern TBI cohorts including TRACK-TBI [3], CENTER-TBI [4], and the PROTIPS cohort studied herein [5]. Three iterations of the IMPACT model have been developed, with the $IMPACT_{Lab}$ model having the best predictive power. As such, we have used the $IMPACT_{Lab}$ model in this study to adjust for baseline risk of unfavorable outcome after msTBI.

Supplemental Methods

Partial Least Square regression with Discriminant Analysis (PLS-DA)

SCFA levels below the lower limit of quantification (eFigure 1) were imputed as half of the lower limit. The association between SCFA levels and outcome was assessed using a two-stage approach (Figure 2). The levels of acetate, propionate and butyrate, the three primary circulating SCFA species, were log_{10}-tranformed and scaled to have zero mean and unit variance. These were then used in Stage 1 as predictors in partial least square discriminant analyses (PLS-DA) with favorable discharge outcome and favorable 6-month outcome as the dependent variables in two separate models. Variable Importance in Projection (VIP) scores were calculated for each SCFA and those with VIP>1 were selected as features for downstream analyses. In Stage 2, log_{10}-tranformed and scaled SCFA levels, for those selected in Stage 1, underwent dimensionality reduction using Principal Component Analysis (PCA). PCA-transformed SCFA levels were clustered using K-means clustering with k=2-10 and the k with the highest silhouette score was selected for further analysis. Cluster validity was assessed using the Adjusted Rand Index and Normalized Mutual Information as well as the between cluster sum-of-squares/total sum-of-squares. SCFA cluster assignment was then used as the independent variable for subsequent analyses that assessed the association with discharge and 6-month functional outcomes.

5-fold Cross Validation of Δpredicted risk.

To reduce optimism and assess the robustness of the Δpredicted risk calculation, we performed internal validation using 5-fold stratified cross-validation. The dataset was partitioned into five approximately equal folds, preserving the proportion of unfavorable outcomes within each fold. For each iteration, a logistic regression model incorporating $IMPACT_{Lab}$ predicted probability and SCFA cluster membership was trained on four folds and evaluated on the held-out fold.

Predicted probabilities from the held-out folds were aggregated to generate out-of-fold predictions for all patients. These cross-validated predictions were used to compute the Δpredicted risk and used for all subsequent reclassification analyses in **eFigure 5**. The baseline $\text{IMPACT}_{\text{Lab}}$ predicted probabilities were treated as fixed and were not refit during cross-validation.

eFigure 1

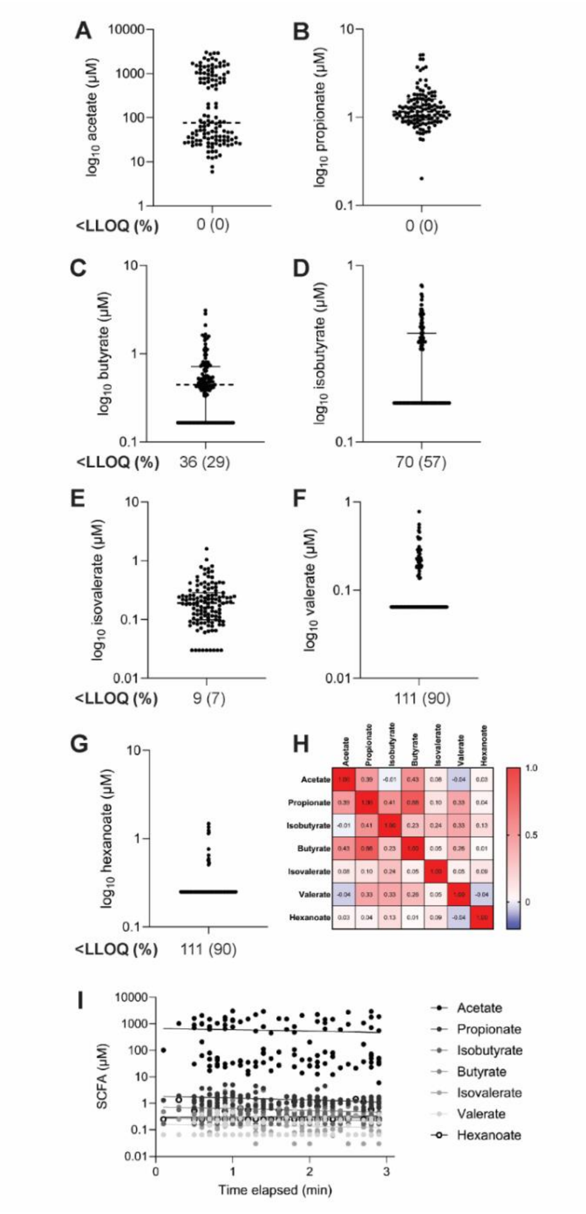

eFigure 1. (A-G) Distribution of each of the seven detected SCFA species. Dashed line indicates median level. The absolute number and percentage of the cohort with the specified SCFA below the limit of quantification (LLOQ) is shown for each panel. SCFA levels below the LLOQ were imputed at half the LLOQ. **(H)** Correlation matrix showing within-subject associations between individual SCFAs. **(I)** No significant association between the levels of individual SCFAs and time elapsed between trauma and sample collection. All slopes of linear regression were not significantly different than zero (corrected p>0.05).

eFigure 2

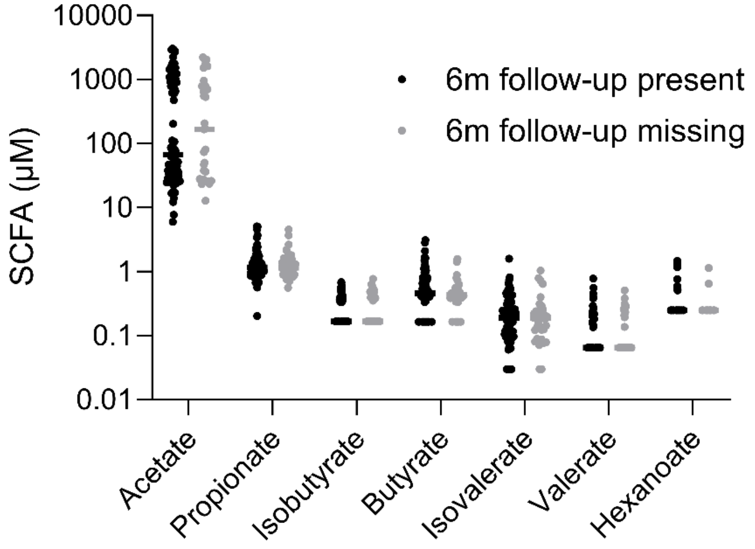

eFigure 2. No differences between SCFA levels in subjects with follow-up at 6 months and those with missing 6 month outcome data. Two-way ANOVA: SCFA: $F_{(1,121)}$ = 54.06, p<0.0001; 6m follow-up missing: $F_{(1,121)}$ = 0.022, p=0.88.

eFigure 3

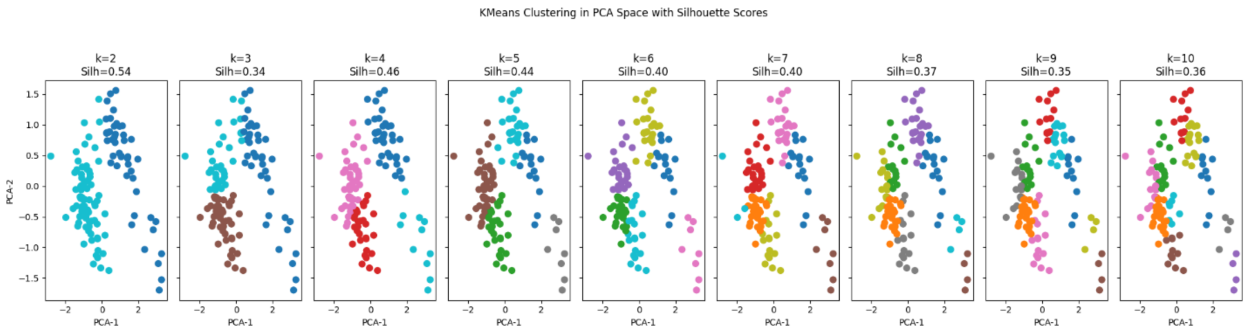

eFigure 3. K-means clustering of the PCA reduction of acetate and propionate for each subject using K=2-10. K=2 had the highest silhouette score and was chosen for further analyses. Each dot represents an individual subject. Different colors within a panel represent cluster.

eFigure 4

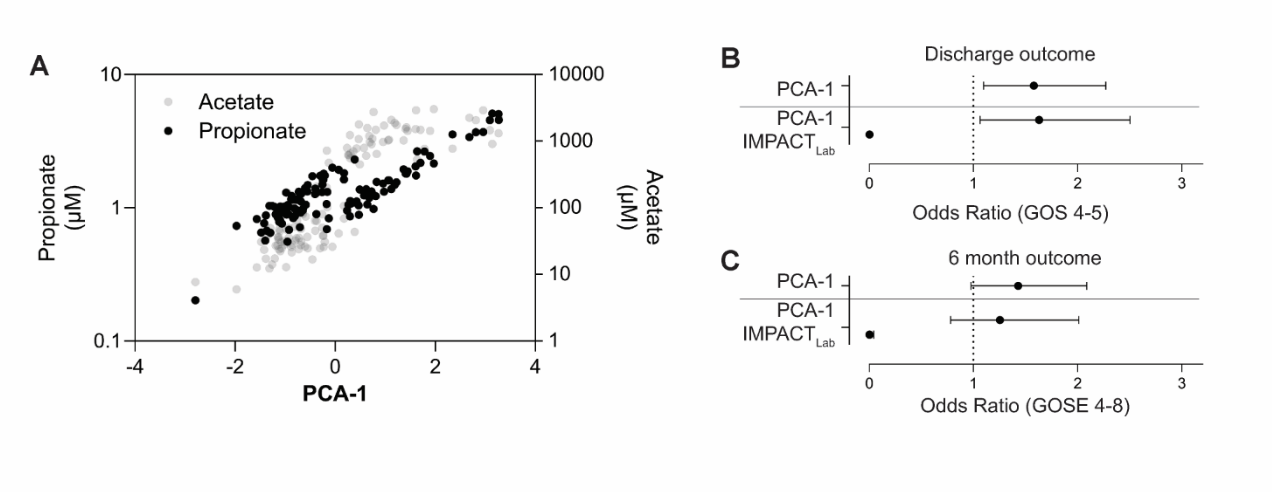

eFigure 4. (A) Correlation between propionate (left y-axis; black), acetate (right y-axis; grey) and PCA-1 (x-axis) from PCA of acetate and propionate from each subject. **(B-C)** Logistic regression using dichotomized **(B)** discharge (GOS 4-5) and **(C)** 6-month outcomes (GOSE 4-8) as the dependent variable. **(B)** Top: univariable logistic regression with PCA-1 revealed a significant association with discharge outcome (OR 1.6 (1.10-2.27); p=0.014). Bottom: Multivariable regression including PCA-1 (OR 1.6 (1.06-2.50); p=0.025) and $IMPACT_{lab}$ unfavorable outcome prediction (OR 0.002 (0.000-0.026); p=0.000) as covariates. **(C)** Top: univariable logistic regression with PCA-1 revealed an association with 6-month outcome (OR 1.4 (0.98-2.09); p=0.066). Bottom: Multivariable regression including PCA-1 (OR 1.3 (0.78-2.01); p=0.35) and $IMPACT_{lab}$ unfavorable outcome prediction (OR 0.003 (0.000-0.042); p=0.000) as covariates.

eFigure 5

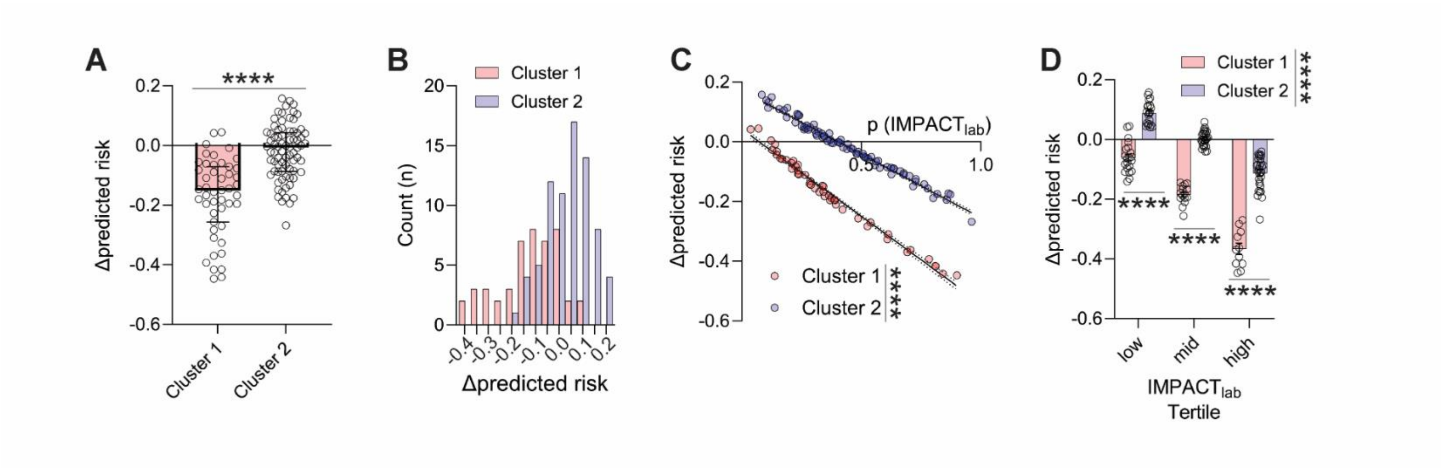

eFigure 5. (A) The change in out-of-fold predicted risk of unfavorable outcome between the outcome prediction model using both SCFA cluster + $\mathrm{IMPACT_{Lab}}$ or using $\mathrm{IMPACT_{Lab}}$ alone (Δpredicted risk) was greater in subjects assigned to SCFA cluster 1. Two-tailed unpaired t test: $p<0.0001$. **(B)** Representation of the data from **(A)** as a histogram of out-of-fold Δpredicted risk highlights the difference between SCFA cluster. **(C)** The relationship between out-of-fold Δpredicted risk and the baseline probability of an unfavorable outcome from the $\mathrm{IMPACT_{Lab}}$ model is different between SCFA clusters. Analyzed using linear regression, which showed that slopes were significantly different between SCFA clusters ($p<0.0001$). **(D)** There was a significant interaction between tertile of baseline probability of unfavorable outcome (based on $\mathrm{IMPACT_{Lab}}$ alone) and out-of-fold Δpredicted risk. Two-way ANOVA: out-of-fold Δpredicted risk x $\mathrm{IMPACT_{Lab}}$ tertile: $F_{(2,117)} = 12.63$, $p<0.0001$. Post-hoc Holm-Sidak testing demonstrated significant differences in Δpredicted risk for each $\mathrm{IMPACT_{Lab}}$ tertile. * $p<0.05$, ** $p<0.01$, **** $p<0.0001$.

1. Murray GD, Butcher I, McHugh GS, Lu J, Mushkudiani NA, Maas AIR, et al. Multivariable Prognostic Analysis in Traumatic Brain Injury Results from The IMPACT Study. J Neurotrauma. 2007;24:329–37. https://doi.org/10.1089/neu.2006.0035

2. Steyerberg EW, Mushkudiani N, Perel P, Butcher I, Lu J, McHugh GS, et al. Predicting Outcome after Traumatic Brain Injury: Development and International Validation of Prognostic Scores Based on Admission Characteristics. PLoS Med. 2008;5:e165. https://doi.org/10.1371/journal.pmed.0050165

3. Yue JK, Lee YM, Sun X, Essen TA van, Elguindy MM, Belton PJ, et al. Performance of the IMPACT and CRASH prognostic models for traumatic brain injury in a contemporary multicenter cohort: a TRACK-TBI study. J Neurosurg. 2024;141:417–29. https://doi.org/10.3171/2023.11.jns231425

4. Dijkland SA, Helmrich IRAR, Nieboer D, Jagt M van der, Dippel DWJ, Menon DK, et al. Outcome Prediction after Moderate and Severe Traumatic Brain Injury: External Validation of Two Established Prognostic Models in 1742 European Patients. J Neurotrauma. 2021;38:1377–88. https://doi.org/10.1089/neu.2020.7300

5. Takegami N, Torres-Espin A, Imagawa Y, Watanabe I, Rowell S, Schreiber M, et al. Evaluating and Updating the IMPACT Model to Predict Outcomes in Two Contemporary North American Traumatic Brain Injury Cohorts. J Neurotrauma. 2024; https://doi.org/10.1089/neu.2024.0158
